# Influence of Persistent Inflammation in Follow-Up Biopsies After Antibody-Mediated Rejection in Kidney Transplantation

**DOI:** 10.3389/fmed.2021.761919

**Published:** 2021-11-12

**Authors:** Gaston J. Piñeiro, Enrique Montagud-Marrahi, José Ríos, Pedro Ventura-Aguiar, David Cucchiari, Ignacio Revuelta, Miquel Lozano, Joan Cid, Frederic Cofan, Nuria Esforzado, Eduard Palou, Federico Oppenheimer, Josep M. Campistol, Beatriu Bayés-Genís, Jordi Rovira, Fritz Diekmann

**Affiliations:** ^1^Department of Nephrology and Kidney Transplantation, Hospital Clinic Barcelona, Barcelona, Spain; ^2^Laboratori Experimental de Nefrologia i Trasplantament (LENIT), Institut d'Investigacions Biomèdiques August Pi i Sunyer (IDIBAPS), Barcelona, Spain; ^3^Medical Statistics Platform, Institut d'Investigacions Biomques August Pi i Sunyer (IDIBAPS), Barcelona, Spain; ^4^Apheresis Unit, Department of Hemotherapy and Hemostasis, Hospital Clínic de Barcelona, Universitat de Barcelona, Barcelona, Spain; ^5^Department of Immunology, Hospital Clc de Barcelona, Universitat de Barcelona, Barcelona, Spain; ^6^Red de Investigación Renal (REDINREN), Madrid, Spain

**Keywords:** kidney transplantation, antibody-mediated rejection, graft failure, follow-up biopsy, microvascular inflammation

## Abstract

**Background:** Despite recent advances in immunosuppression treatment, antibody-mediated rejection (ABMR) remains the leading cause of kidney graft loss. Information about prognostic markers and the efficacy of treatment is scarce.

**Methods:** Retrospective study with kidney recipients diagnosed an active ABMR from January 1, 2004 to December 31, 2019 to explore the influence of persistent inflammation in follow-up biopsies on graft survival after ABMR treatment.

**Results:** About 116 patients were included. Active ABMR were treated with a combination of plasma exchange (PE), intravenous immunoglobulin (IVIg), rituximab, and steroids. At 6 months of treatment, 63 (54.3%) patients presented a stabilization or improvement in kidney-graft function. The effectiveness varied depending on the timepoint of the presentation between transplantation and rejection, which is lower for those with late ABMR (63 vs. 21% for early vs. late ABMR, respectively). Ninety patients (77%) underwent a control biopsy after ABMR treatment, from which 46 (51%) responded to the treatment. Microvascular inflammation (MVI) persisted in 64 (71%) biopsies, whereas tubulitis persisted in 17 (19%) biopsies. Death-censored graft survival at 1 year was significantly lower in patients with persistent MVI (86% vs. 95% without persistent MVI, *P* = 0.002), or with persistent tubulitis (44% vs. 66% without tubulitis, *P* = 0.02). In the Cox Regression analysis, the persistence of MVI [hazard ratio (HR), 4.50 (95%CI, 1.35–14.96), *P* = 0.01] and tubulitis [HR 2.88 95%CI (1.24–6.69), *P* = 0.01) in follow-up biopsies significantly increased the risk of graft failure.

**Conclusion:** Persistent inflammation in follow-up biopsies after ABMR treatment was associated with an increased risk of graft loss, even without meeting Banff rejection criteria.

**Study Registration:** Agencia Española de Medicamentos y Productos Sanitarios (AEMPS): 14566/RG 24161. Study code: UTRINM-2017-01.

## Introduction

Along with the improvement of immunosuppression strategies, antibody-mediated rejection (ABMR), especially chronic active ABMR, has been increasing as the leading cause of late kidney graft failure (1, 2). Also, ABMR has been linked with worse patient survival (3–5). However, despite the clinical relevance of ABMR, there is no specific treatment for ABMR approved by the Food and Drug Administration (FDA) or the European Medicines Agency (EMA). Plasma exchange (PE), intravenous immunoglobulin (IVIg), and corticosteroids constitute the most common strategy for ABMR treatment and are considered the standard of care for many kidney transplant societies. Also, rituximab is widely used as off-label to prevent and treat ABMR without any clear evidence of efficacy (6–9). Available information about its effectiveness and treatment complications is scarce; this makes it difficult to make decisions, especially when reassessing a kidney recipient after ABMR treatment. In this sense, the information derived from follow-up biopsies after ABMR treatment could be potentially useful when assessing ABMR prognosis.

Herein, we analyze the impact of PE, IVIg, steroids, and rituximab treatment after ABMR on kidney graft and we revise the impact of this treatment through follow-up biopsies in a cohort of patients after ABMR treatment to determine a prognostic marker of response, focusing on histological inflammation.

## Materials and Methods

### Study Design and Patient Population

We performed a longitudinal single-center retrospective study, which included kidney recipients diagnosed with ABMR, according to the Banff 2017 classification. Concretely, we have identified kidney recipients who received a treatment for ABMR from January 1, 2004 to December 31, 2019 (including a combination of PE, IVIg, and rituximab) in the database of Renal Transplant Unit at Hospital Clinic de Barcelona; then biopsies at ABMR diagnosis were reanalyzed according to the criteria specified by Banff (2017). Recipients who received a multivisceral transplant, and those with transplant glomerulopathy (TG) in the initial biopsy, cg ≥ 1 in the Banff histopathological classification, were excluded (10).

Demographic, clinical, biochemical, histopathological, and immunological data were evaluated for both the donor and the recipient. Clinical characteristics, maintenance immunosuppression, and ABMR treatment were analyzed at the diagnosis and follow-up period. Charlson comorbidity index (CCI) was also assessed at ABMR diagnosis (11).

Infections that required hospitalization at least 48 h within the first year after ABMR diagnosis were recorded and described in relation to clinical variables.

The study was performed according to the Declaration of Helsinki principles and approved by the Hospital Research Ethics Committee. Study registration: Agencia Española de Medicamentos y Productos Sanitarios (AEMPS): 14566/RG 24161. Study code: UTRINM-2017-01.

### Antibody-Mediated Rejection Diagnosis

The decision to perform a renal biopsy and patient treatment was based on the clinical judgment at ABMR diagnosis, including biopsies due to impaired renal function and protocol biopsies (at 3 or 12 months after kidney transplantation). Active ABMR was diagnosed and categorized according to the Banff criteria of 2017 (10). The day when the biopsy was diagnosed was considered as the date of active ABMR diagnosis. Immunologically, donor-specific antibodies (DSAs) were tested using Single Antigen Bead Test (LIFECODES^®^ Single Antigen, Immucor, Georgia, US). In our Center, the Single Antigen Beads Test has been used since 2001. However, the criteria to consider an allele positive (the MFI over 1,500 and 4 times higher than the Lowest Reactive Antigen of the same locus) changed in 2017. Since 2017, the criterion is that the MFI is >750 and the cut-off for the ratio [MFI/LRA (Lowest Ranked Antigen)] is specific for each individual bead (12).

### Definition of Outcomes

Our primary outcome was death-censored kidney graft survival at 1 year after ABMR diagnosis and at the follow-up period, between patients with and without persistent inflammation in the control biopsy, either microvascular (MVI, defined as g + ptc ≥ 1) or tubular (*t* ≥ 1). Follow-up biopsies after ABMR treatment were performed according to the physician's criteria. Secondary outcomes were defined as kidney graft function at 6 months from the ABMR treatment and kidney graft function at the last follow-up. Kidney graft function was assessed by serum creatinine (SCr), estimated glomerular filtration rate (eGFR, according to the Chronic Kidney Disease Epidemiology Collaboration equation), and urine protein to creatinine ratio (UPCR) (13, 14). Early ABMR was defined as that which occurred within 6 months from the kidney transplant, while late ABMR was defined as that occurring after 6 months from the kidney transplant (15).

Patient survival was defined as the last day of follow-up or the date of death. Kidney graft failure was defined as one of the following: return to dialysis or re-transplantation. Response to ABMR treatment was defined as improvement or stabilization of eGFR at 6 months compared to eGFR at ABMR diagnosis.

PE was performed in Cobe Spectra or Spectra Optia separators (Terumo BCT, Lakewood, CO, USA) using 5% albumin (Albutein® 5%, Grífols, Spain) as a replacement solution. One plasma volume was exchanged in each session (16).

### Statistical Analysis

Data are presented as mean (SD) for parametric variables, and median [interquartile range (IQR)] for the non-parametric ones. The corresponding tests used were the *t*-test, McNemar Test, Wilcoxon test, Chi-Square, and ANOVA as appropriate.

Kaplan–Meier was used to estimate graft survival and compared using the log-rank test. For the survival analysis throughout the follow-up, we used the Cox regression model, and the logistic regression model was used for the 1-year survival analysis using IBM SPSS Statistics 26.0 (SPSS, Inc; Chicago, Illinois) software for Windows. All the tests were two-tailed, and the significance level was defined as a *P*-value < 0.05.

## Results

### Characteristics of Baseline Donor and Recipient

**Figure 1:**
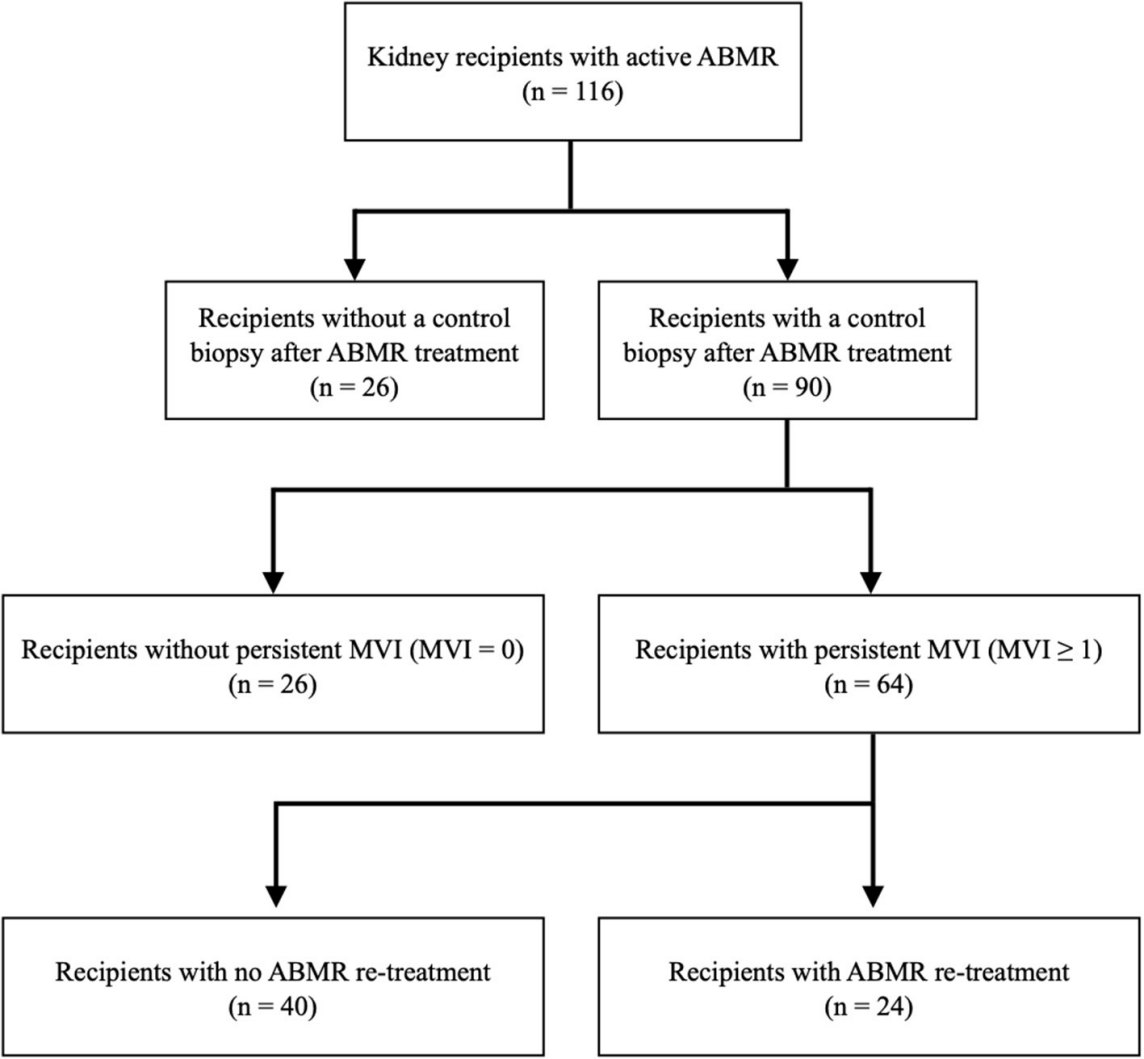
Flowchart of the included patients. ABMR, antibody-mediated rejection; MVI, microvascular inflammation.

From January 2004 to December 2019, 116 kidney transplant recipients were diagnosed with an active ABMR (Figure 1). In 97 patients (83.6%), the biopsy was performed by indication due to impaired renal function, while the other 19 patients (16.4%) underwent a protocol biopsy at 3 or 12 months after transplantation. Table 1 summarizes demographical and clinical data at ABMR diagnosis. Most of them were men (59.5%), with a mean age at the rejection of 50.8 ± 14 years, and a median follow-up from ABMR diagnosis of 33.5 [62.7] months. The median duration of dialysis was 4 [4] years. About 76% of the donors were deceased donors. Up to 48% of patients had received a previous kidney transplant, and 14% presented a positive cytometry crossmatch at transplantation.

**Table 1:** Demographic and clinical characteristics at diagnosis of antibody-mediated rejection (ABMR).

**Donor**	**Patients**
	**(***n*** = 116)**
Age (years)	54.67 ± 14.29
Gender (male)	59 (50.9)
DDKT	83 (71.6)
DCD	12 (10.3)
Recipient age (years)	50.8 ± 14
Recipient gender (male)	69 (59.5%)
Dialysis vintage (years)	4 (4)
Dialysis modality	
Preemptive	19 (16.4)
Hemodialysis	88 (75.9)
Peritoneal dialysis	9 (7.7)
Hypertension (yes)	88 (75.9)
DM (yes)	20 (17.2)
Vasculopathy (yes)	19 (16.4)
HCV (yes)	29 (25)
ESKD etiology	
ADPKD	17 (14.7)
Urological	29 (25)
Glomerulonephritis	26 (22.4)
Diabetic nephropathy	12 (10.3)
Nephroangiosclerosis	7 (6)
Unknown	25 (21.6)
Previous KT (any)	56 (48.3)
HLA mismatches	4.1 ± 1.55
ABO incompatibility (yes)	16 (13.8)
Pre-transplant DSAs (any)	30 (25.8)
DSAs at ABMR diagnosis (any)	73 (62.9)
PRA > 50% (yes)	18 (15.5)
CF-CM at KT (positive)	17 (14.65)
Luminex at KT (positive)	
Class I	39 (33.6)
Class II	44 (37.9)
Induction Immunosuppression (yes)	112 (96.6)
Basiliximab	39 (33.6)
Rituximab	11 (9.5)
Thymoglobulin	68 (58.6)
Immunosuppression maintenance	
Tacrolimus	79 (68.1)
mTORi	38(32.8)
Mycophenolate	89 (76.7)
Steroids	97(83.6)
Characteristics of rejection at ABMR diagnosis	
Cellular rejection	16 (13.8)
Time from KT to ABMR (days)	27.5 [287.7]
ABMR <6 months after KT (yes)	82 (70.7)
Need for dialysis at ABMR (yes)	32 (27.6)
ABMR treatment at diagnosis (yes)	116 (100)
Plasma exchange	111 (95.7)
IVIg	111 (95.7)
Rituximab	101 (87,1)
Corticosteroids	116 (100)

*Data are mean ± SD, median [IQR] or n (%), unless otherwise specified. ABMR, Antibody-mediated rejection; KT, kidney transplantation; HCV, hepatitis C virus; DM, Diabetes Mellitus; ESKD, end-stage kidney disease; ADPKD, autosomal dominant polycystic kidney disease; HLA, human leucocyte antigen; DSAs, donor-specific antibodies; PRA, panel reactive antibodies; CF-CM, flow cytometry crossmatch; mTORi, mTOR inhibitors; DDKT, deceased donor kidney transplantation; DCD, donor after cardiac death; IVIg, intravenous immunoglobulin*.

Regarding the characteristics of rejection (Table 1), the median time from transplantation to active ABMR was 27.5 [287.7] days, with 70.7% (82) of the ABMR episodes diagnosed within 6 months after transplantation and which were considered as early ABMR [median of 16 (21.5) days from transplant]. Twenty-seven of the patients required dialysis after the diagnosis of ABMR.

### Response to Treatment and Survival

All patients were treated for ABMR with a combination of corticosteroids, PE, IVIg, and rituximab. The active ABMR treatment protocol consists of a combination of five sessions of PE, IVIG 200 mg/kg every 2 PE, and two rituximab doses. However, in this cohort, 15 patients (12.9%) were not treated with rituximab by concerns of the treating physician because of a perception of increased risk of infections. Also, five patients (4.3%) did not receive PE treatment for problems related to vascular access. One plasma volume was exchanged in each session with a median of 5 [1] sessions.

The global response to ABMR treatment was 54.3%, with a significant increase in eGFR at 6 months after treatment and at the end of the follow-up period (*P* = 0.003). Table 2 summarizes the changes in eGFR. Patients with an early ABMR [median 16 (21.5) days] have a better response than those with a late ABMR [median 25.9 (40) months], 67 vs. 23.5% for early and late ABMR, respectively, odds ratio (OR) 0.15 [95% CI 0.06–0.38], *P* < 0.001. Overall graft failure at 1 year and throughout the follow-up was 32.8 and 38.8%, respectively. Death-censored graft failure for the same time points was 25.9 and 31%, respectively. The presence of DSA at diagnosis was not associated with worse graft survival (*P* = 0.15).

**Table 2:** Creatinine, estimated glomerular filtrate, and proteinuria after ABMR diagnostic.

	**At ABMR diagnosis** **(N =116)**	**At 6 months** **(***N*** = 98)**	**At follow up** **(***N*** = 63)**	***P*** value*
SCr (mg/dL)	3.67 ± 1.97	2.31 ± 1.49	2.3 ± 1.14	<0.001
eGFR (mL/min)	25.21 ± 16.3	37.63 ± 18.9	36.9 ± 20	0.003
Proteinuria (mg/g)	585 [980.5]	482 [1070]	437 [1194.5]	0.69

* *Respect to baseline. ABMR, antibody-mediated rejection; SCr, serum creatinine; eGFR, estimated glomerular filtration rate*.

The treating physicians decided follow-up biopsy after ABMR treatment in 90 patients. Demographic, clinical, biochemical and immunological characteristics from patients with and without follow-up biopsy have shown in Supplementary Table 1, whereas histological parameters in biopsy at ABMR diagnosis are presented in Supplementary Table 2. The prevalence of living donors, previous kidney transplants, and human leukocyte antigen (HLA) sensitization were higher in the follow-up of patients who underwent biopsy. There were no significant differences in death-censored graft survival, where *P* = 0.31 between patients with or without follow-up biopsy.

Histopathological findings at ABMR diagnosis and follow-up biopsies are summarized and compared in Table 3.

**Table 3:** Banff histopathological findings at diagnostic and follow-up biopsies.

	**At diagnosis** **(***n*** = 116)**	**Follow-up biopsy** **(***n*** = 90)**	***P*** **value**	**Resolution/Presence** **at follow-up (n/n)**
g	0.84 ± 0.95	0.88 ± 1.06	0.98	17 /45
ptc	1.43 ± 0.94	0.99 ± 1.01	0.0013	30/52
MVI (g+ptc)	2.27 ± 1.35	1.87 ± 1.78	0.02	17/64
t	0.45 ± 0.82	0.24 ± 0.57	0.03	18/16
i	0.68 ± 0.91	0.46 ± 0.77	0.01	17/24
ti	0.53 ± 0.8	0.53 ± 0.78	0.4	
v	0.2 ± 0.56	0.03 ± 0.23	0.002	
mm	0.09 ± 0.31	0.2 ± 0.56	0.075	
ah	0.31 ± 0.7	0.42 ± 0.72	0.21	
cg	0	0.19 ± 0.47	<0.001	-/14
ci	0.65 ± 0.76	1.07 ± 0.88	<0.001	5/65
ct	0.59 ± 0.73	1.06 ± 0.83	<0.001	6/79
cv	0.61 ± 0.75	0.81± 0.85	0.16	
C4d	95 (82)	65 (56)	<0.001	10/63
IFTA	0.54 ± 0.69	0.81 ± 0.98	0.009	

*Results are shown as mean ± SD for quantitative and qualitative variables. Qualitative variables, as C4d, are shown in absolute number and the percentage in brackets (% positive). g, glomerulitis; ptc, peritubular capillaritis; MVI, microvascular inflammation; t, tubulitis; i, interstitial inflammation; ah, arterial hyalinosis; cg, transplant glomerulopathy; ci, interstitial fibrosis; ct, tubular atrophy; IFTA, interstitial fibrosis + tubular atrophy; cv, vascular fibrous intimal thickening*.

The presence of tubulitis at ABMR diagnosis was associated with an increased risk of graft loss at 1 year (OR 1.79 [95% CI 1.05–3.06], *P* = 0.03) and at follow up [HR 2.10 (95% CI 1.04–4.26), *P* = 0.04]. The combination of interstitial fibrosis and tubular atrophy (IFTA) and the coexistence of a T Cell-mediated rejection (TCMR) were associated with an increased risk of graft loss at follow-up [HR 1.62 (95% 1.09–2.40), *P* = 0.02 and HR 2.48 (95% CI 1.07–5.76), *P* = 0.03 for IFTA and TCMR, respectively; Table 4A].

**Table 4:** Analysis for death-censored graft failure.

**(A) Biopsy at ABMR diagnosis** **(***n*** = 116)**	**At 1 year**		**At follow-up**	
	**OR (95% CI)**	***P*** **value**	**HR (95% CI)**	***P*** **value**
Glomerulitis ≥ 1	0.73 (0.39–1.36)	0.31	0.92 (0.64–1.34)	0.68
Peritubular capillaritis ≥ 1	1.84 (1.01–3.35)	0.047	1.76 (0.68–4.56)	0.25
MVI ≥ 1	2.04 (0.54–7.66)	0.29	1.19 (0.93–1.51)	0.16
Tubulitis ≥ 1	1.79 (1.05–3.06)	0.03	2.10 (1.04–4.26)	0.04
Vascular inflammation ≥ 1	0.72 (0.22–2.32)	0.57	0.76 (0.23–2.50)	0.65
TMA ≥ 1	1.27 (0.14–11.6)	0.83	0.82 (0.11–6.02)	0.85
Total inflammation ≥ 1	1.32 (0.46–3.85)	0.6	1.07 (0.66–1.74)	0.78
ATN (yes)	0.49 (0.13- 1.84)	0.29	0.80 (0.38- 1.67)	0.55
Positive C4d (yes)	0.95 (0.25–3.69)	0.94	1.23 (0.42–3.57)	0.71
IFTA (yes)	3.10 (1.00–9.64)	0.05	1.62 (1.09–2.40)	0.02
Positive DSA	0.68 (9.20–2.30)	0.53	0.74 (0.36–1.5)	0.4
Cellular rejection (yes)	1.50 (0.39–6.20)	0.46	2.48 (1.07–5.76)	0.03
**(B) Follow-up biopsy** **(*****n*** **= 90)**	**At 1 year**		**Throughout follow-up**	
	**OR (95% CI)**	***P*** **value**	**HR (95% CI)**	***P*** **value**
Glomerulitis ≥ 1	1.20 (0.37–3.89)	0.76	2,54 (1.17–5.49)	0.02
Peritubular capilaritis ≥ 1	4.83 (1.00–23.2)	0.049	2.99 (1.27–7.07)	0.01
MVI ≥ 1	1.73 (0.52–5.76)	0.37	4.50 (1.35–15.0)	0.01
Tubulitis ≥ 1	1.85 (0.79–4.31)	0.15	2.88 (1.24–6.69)	0.01
Vascular inflammation ≥ 1	1.09 (0.21–5,60)	0.99	0.85 (0.26–2.83)	0.79
TMA (yes)	11.41 (2.54–51.3)	0.001	3.70 (1.50–9.15)	0.005
Total inflammation ≥ 1	3.14 (0.93–10.6)	0.06	4.56 (1.53–13.6)	0.006
ATN (yes)	8.11 (1.72–38.2)	0.008	4.56 (1.53–13.6)	0.006
Positive C4d (yes)	2.34 (0.48–11.4)	0.29	1.77 (0.60–5.19)	0.29
TG (yes)	4.72 (1.27–17.6)	0.02	2.24 (1.33–3.77)	0.002
IFTA (yes)	1.43 (0.81–2.52)	0.21	1.62 (1.14–2.32)	0.008
Positive DSA (yes)	0.63 (0.16–2.47)	0.75	0.72 (0.32–1.62)	0.43
Cellular rejection (yes)	1.60 (0.22–3.00)	0.63	3.00 (1.06–8.80)	0.039

*Univariate analysis. Results are shown as mean ± SD. MVI, microvascular inflammation; TMA, thrombotic microangiopathy; ATN, acute tubular necrosis; TG, transplant glomerulopathy; IFTA, interstitial fibrosis and tubular atrophy; DSA, donor-specific antibodies*.

### Impact of Persistent Inflammation in Follow-Up Biopsies

A follow-up biopsy after ABMR treatment was performed in 90 patients (77.6%), with a median time from the treatment of 2 [4] months. In general, there was a significant decrease in inflammation and an increase in chronicity parameters between the biopsy at diagnosis and the follow-up biopsy (Table 3).

Although 46 out of 90 rebiopsied patients (51%) had responded clinically to ABMR treatment, about 64 (71%) follow-up biopsies presented a persistent MVI ≥ 1, and in 45 (50%), MVI ≥ 2 was detected. Twenty-six out of 46 patients with improvement in graft function had persistence of MVI ≥ 1. There were no statistically significant differences in baseline characteristics between patients with and without persistent MVI (Supplementary Table 3). There were also no significant histological differences in the biopsy at ABMR diagnosis between patients with and without the persistence of microvascular inflammation, except for a greater severity of MVI in patients with persistent MVI (Supplementary Table 4).

The time between kidney transplantation and graft biopsy was similar between patients with and without inflammation persistence in the follow-up biopsies (*P* = 0.23). The presence of DSA or the DSA title was similar between the two groups (*P* = 0.09). The presence of preformed DSA was associated with early rejection (*P* = 0.007; OR 5.1, CI95% 1.4–18.1). However, *de-novo* DSA was not associated with a higher risk of graft loss or persistence of MVI in the follow-up biopsies.

**Figure 2:**
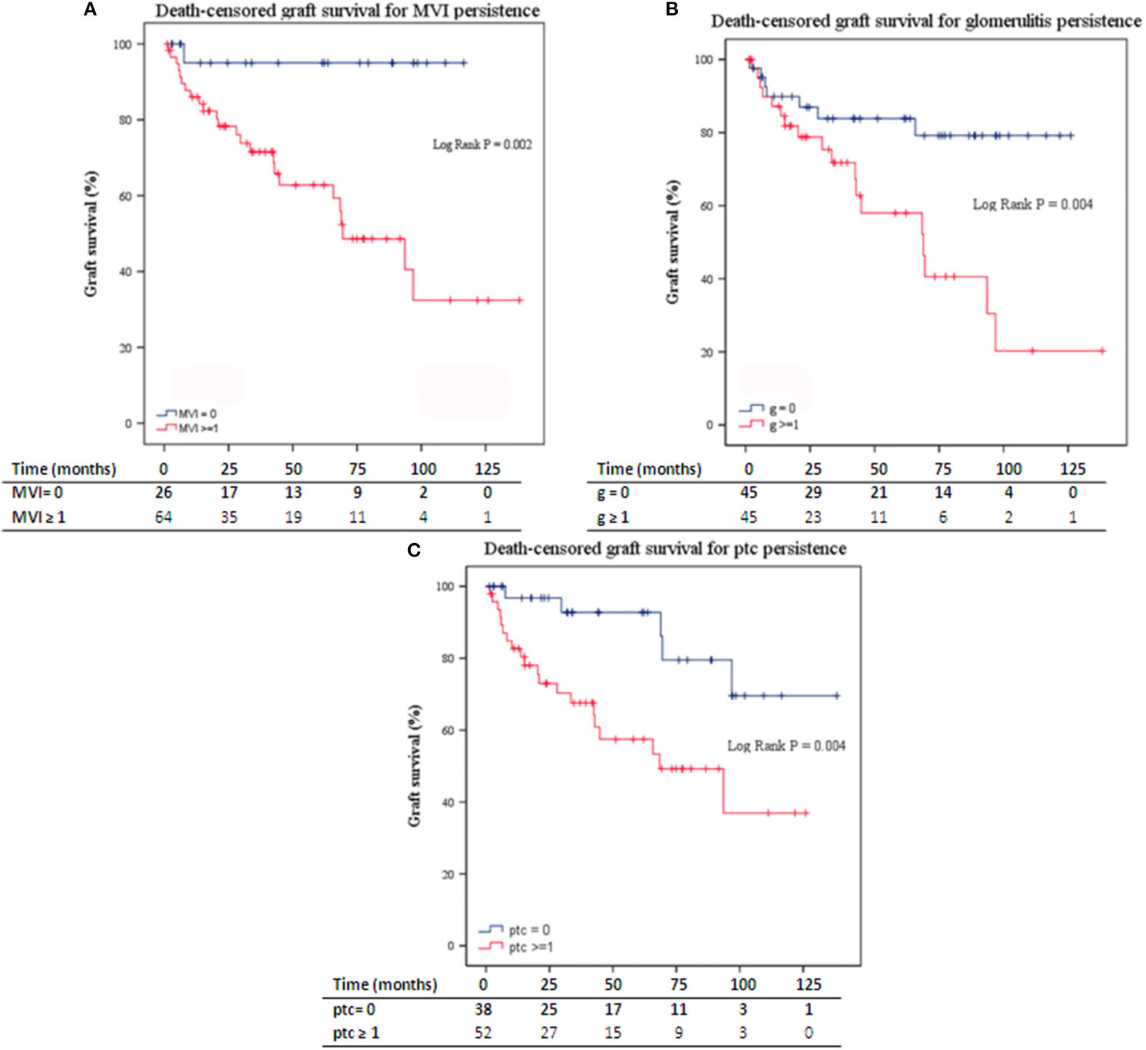
**(A)** Death-censored graft survival for MVI persistence in follow-up biopsies. **(B)** Death-censored graft survival for the persistence of glomerulitis in follow-up biopsies. **(C)** Death-censored graft survival for the persistence of ptc in follow-up biopsies. ABMR, antibody-mediated rejection; MVI, microvascular inflammation; ptc, peritubular capillaritis. Numbers along the x-axis are the numbers of patients remaining in the risk set at each time point.

Death-censored kidney graft survival was significantly lower in those patients with persistent MVI (Figure 2A; Log-Rank, *P* = 0.002). Patients with glomerulitis (g) or peritubular capillaritis (ptc) had significantly lower death-censored kidney graft survival than patients without g or ptc at follow-up biopsy (Figures 2B,C).

In the Cox Regression analysis, the persistence of acute inflammatory lesions in the follow-up biopsies [g, ptc, MVI, tubulitis, acute tubular necrosis (ATN), and thrombotic microangiopathy (TMA)], significantly increased the risk of kidney graft loss throughout the follow-up (Table 4B). Also, the presence of TMA and ATN was associated with 1-year graft loss.

Here a multivariate analysis has many statistical limitations due to the number of events. Therefore, a bivariate analysis was performed instead of a multivariate analysis. After the adjusted analysis for the different histological characteristics, the persistence of MVI in the follow-up biopsies remains a risk factor for graft loss (Supplementary Tables 5, 6).

**Figure 3:**
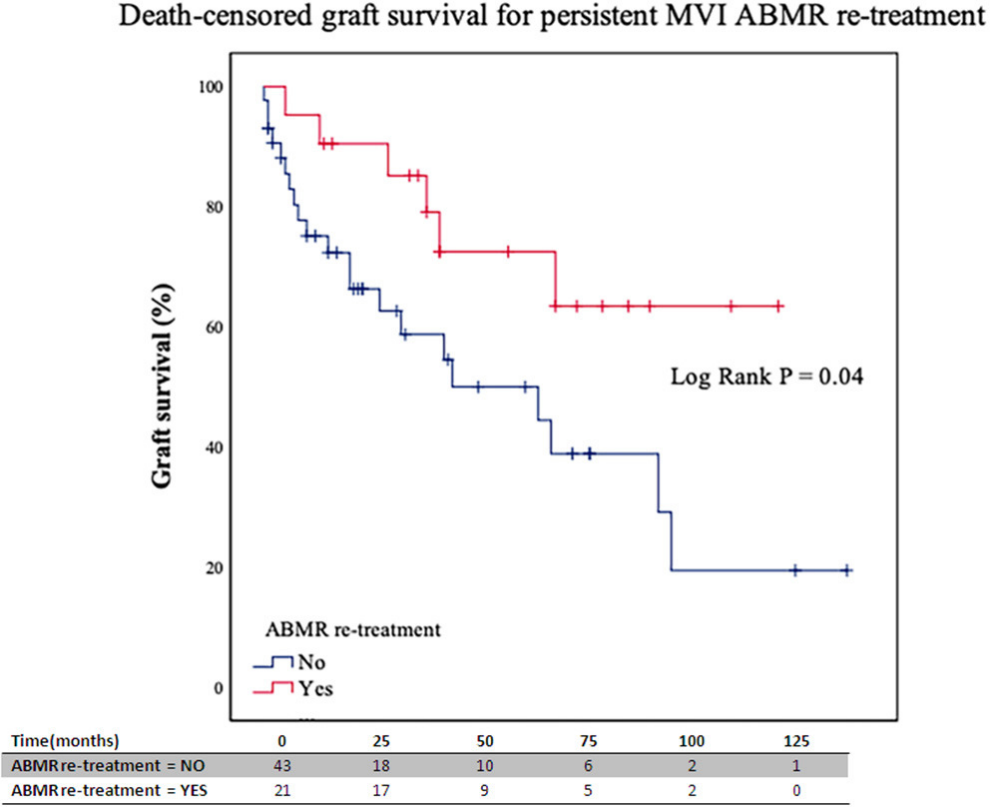
Death-censored graft survival for retreated and non-retreated patients with MVI persistence in follow-up biopsies. ABMR, antibody-mediated rejection; MVI, microvascular inflammation. Numbers along the x-axis are the numbers of patients remaining in the risk set at each time point.

There was no specific retreatment protocol, and therapy was the decision of the treating physicians. Twenty-four out of 64 (37.5%) patients with persistent MVI in follow-up biopsies were retreated with a combination of rituximab (5 patients) and PE (19 patients). Death-censored kidney graft survival was significantly higher for those patients with persistent MVI in control biopsy who were retreated (95 and 73% vs. 75 and 50% at 1 and 5 years after retreatment, respectively, *P* = 0.04; Figure 3). In the Cox regression analysis, ABMR retreatment for those patients with persistent MVI was associated with a lower risk of graft loss [HR 0.40 (95% CI 0.16–0.99), *P* =0.048].

**Figure 4:**
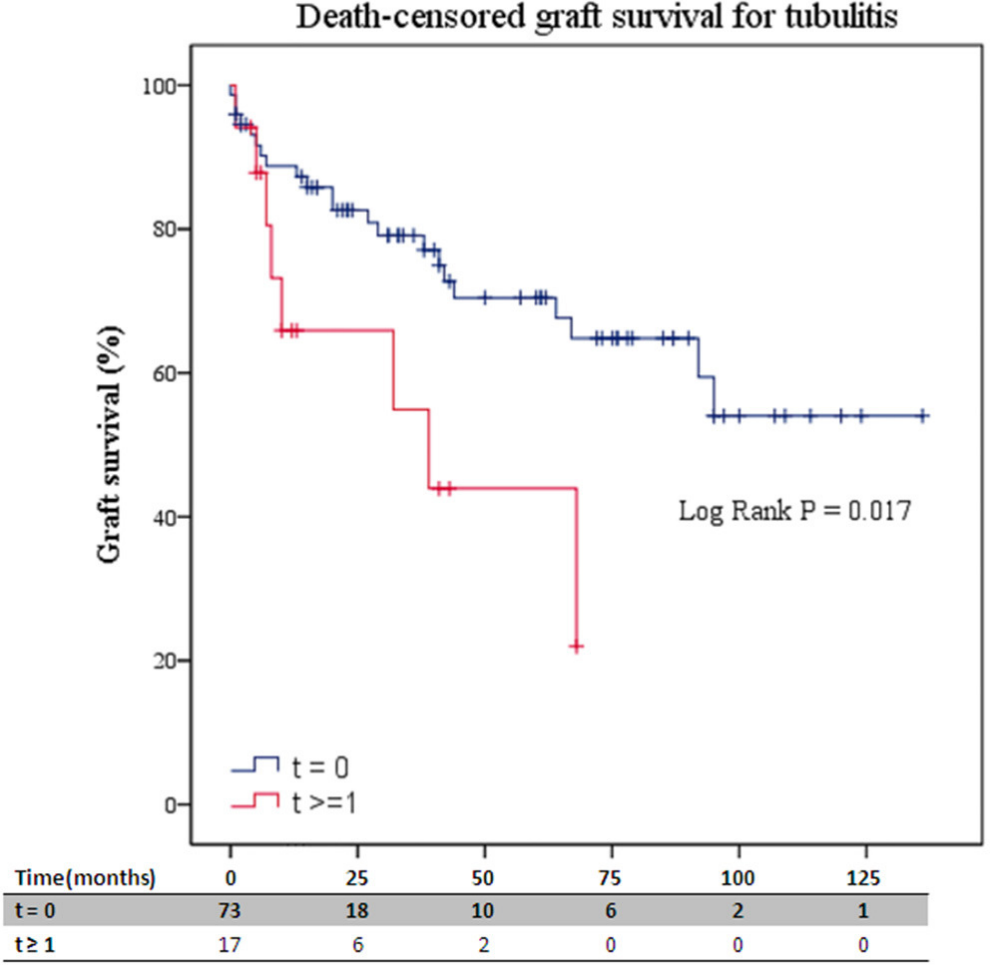
Death-censored graft survival for tubulitis presence in follow-up biopsies. ABMR, antibody-mediated rejection; t, tubulitis. Numbers along the x-axis are the numbers of patients remaining in the risk set at each time point.

Presence of tubulitis (t) was observed in 19% (17) of follow-up biopsies, from which 13 had t grade ≥ 1 without meeting the active or chronic TCMR diagnosis criteria. Patients with *t* ≥ 1 in follow-up biopsies exhibited worse kidney graft survival rates at 1 year (66 and 44%, *P* = 0.02) (Figure 4). Moreover, the persistence of tubulitis in follow-up biopsies significantly increased the risk of kidney graft failure [HR 2.88 (95%CI 1.24–6.69), *P* = 0.01; Table 4B and Supplementary Table 6].

### Chronic Lesions in Follow-Up Biopsies

The presence of TG [HR 2.24 (95% CI 1.33–3.77), *P* = 0.002], and IFTA [HR 1.62 (95% CI 1.14–2.32), *P* = 0.008) in follow-up biopsies were associated with an increased risk of kidney graft failure at follow-up. TG was also associated with an increased risk of 1-year kidney graft loss [OR 4.72 (95% CI 1.27–17.61), *P* = 0.02; Table 4B).

Given the different responses to treatment according to the time from transplantation to rejection, we analyzed the histological characteristics associated with graft loss adjusted for time to rejection (early/late) in the biopsy at ABMR diagnosis and in the follow-up biopsy. Results were shown in Supplementary Table 7. In the time-adjusted analysis, the presence of tubulitis or concomitant cellular rejection in the biopsy at ABMR diagnosis was associated with graft loss. In the follow-up biopsy, the persistence of MVI> 1, ATN, or TMA and the appearance of chronic lesions (cg, IFTA) were associated with graft loss.

### Infectious Complications

During the year after ABMR treatment, 101 infections required hospital admission at least 48 h in 57 patients, which were supposed to be the infection rate of 0.87 infections/treated patient. The presence of the comorbidities of the recipients was associated with an increased risk of infectious complications with admission requirement, with CCI ≥ 4 associated with an increased risk of infections [OR 4.2 (95%CI 1.79–9.81), *P* = 0.01].

The potential association between the total immunosuppression received and infections was analyzed. The following items were not associated with the development of infections: time from transplantation to ABMR treatment (*P* = 0.72), induction with thymoglobulin (*P* = 0.31), induction with rituximab (*P* = 0.7), any previous kidney transplantation (*P* = 0.58), and ABMR re-treatment (*P* = 0.31).

## Discussion

We analyzed short- and long-term kidney graft outcomes of recipients with persistent inflammation after active ABMR treatment, comparing them with those without inflammation on follow-up biopsies. In a cohort of 116 kidney recipients diagnosed with an active ABMR, 90 patients underwent a control biopsy after ABMR treatment. We observed persistent inflammation in the follow-up biopsies after ABMR treatment associated with a higher kidney graft failure rate. These findings suggest that persistent inflammation after an active ABMR had a prognostic value in kidney graft outcomes despite not strictly meeting any of the Banff categories. Moreover, they reinforce the importance of follow-up biopsies after ABMR treatment to guide the therapeutic decision-making of transplant physicians.

Active ABMR constitutes one of the most frequent complications in kidney transplantation (1, 4). Nevertheless, despite the therapeutic advances in immunosuppressive treatment during the last years, ABMR continues to be the most common cause of kidney graft loss (1, 4, 5). At present, the most common strategy adopted by many transplant centers for active ABMR treatment comprises a combination of PE and IVIgs (5, 8, 17), which is considered by the transplant community as the standard of care despite the lack of a solid evidence which supports its usefulness. Also, rituximab is widely used, although there are no solid studies that have evaluated its efficacy (3, 5–7, 18). The degree of effectiveness of the treatment may vary according to the time of the treatment being considered. In this cohort, the current treatment for ABMR has relatively low efficacy, with only 54.3% of patients reaching a stabilization or improvement in kidney graft function of 6 months after treatment. Moreover, the effectiveness markedly varies depending on the timepoint presentation between transplantation and rejection; about 63% of patients with an early ABMR exhibited a significant response, while only 24% of those with a late ABMR responded to ABMR treatment. This observation is consistent with that reported in previous studies (19, 20) and suggests different immunological pathways between both types of acute antibody-mediated rejection (aABMR). Thus, it has been suggested that early rejection is associated with a donor presensitization, an observation that agrees with our results. Moreover, some studies have found an association within the DSA class (I or II) and the aABMR temporality, although these findings remain controversial (19, 20). These results contrast with ours, since we did not find any significant association with early/late aABMR and the DSA class. Nevertheless, it has to be noticed that we only included patients with an aABMR and those with a chronic ABMR were excluded.

Another important issue derived from the most used schemes for ABMR treatment is the severe complications which are derived from these immunosuppression regimens, especially infections and *de novo* neoplasms (1, 4, 5). In this study, we observed a high incidence of infections which required hospital admission for at least 48 h (0.87 infections/treated patient). Importantly, the risk of infection was associated with the comorbidities of the patients having a CCI ≥ 4, which is an independent risk factor for new onset of infections. It should be noted that most of the patients received treatment with rituximab, which has been associated with an increased risk of infections in postkidney transplantation, although there are no data from randomized studies which clearly demonstrate the association between rituximab and infections (21–23).

Therefore, since active ABMR treatment is linked to high morbidity and mortality in kidney transplant recipients, clinical, analytical, and histological prognostic markers are essential to identify those patients who will potentially benefit from those intensive immunosuppressive strategies to avoid futile interventions associated with a high rate of complications and poor benefit (3–5). In this sense, some studies have tried to validate scores based on a combination of clinical and histological variables both at the diagnosis and in the subsequent patient reassessment after rejection treatment (5).

In the absence of conclusive data on the efficacy of retreatment, and considering the high rate of infections in patients with comorbidities, we would propose that such patients should not be retreated. Perhaps the development of new treatments with a better safety profile could be considered in these patients.

A controversial aspect that has not been solidly evaluated in previous studies is the usefulness of performing control graft biopsies after ABMR treatment, usually indicated in a heterogeneous manner and according to individual physician decision (6, 24, 25). In the present study, a control biopsy was performed in 78% of the included patients, of which 53% had presented a satisfactory response to ABMR treatment. Remarkably, we observed that 71% of the rebiopsied patients persisted with MVI ≥ 1. The negative impact of MVI ≥ 1 has been evidenced in previous studies as a potential predictor of the subsequent development of ABMR. However, only one of these studies has evaluated the specific influence of ptc in follow-up biopsies, where it was associated with a higher risk of graft loss (5, 24, 25). Other studies have demonstrated that inflammation in early protocol biopsies is associated with fibrosis progression and development *of de novo* DSAs (26).

More importantly, a key element from our results is that most of the rebiopsied patients had presented an improvement in graft function after the active ABMR treatment, and even in them, the persistence of MVI ≥ 1 was associated with worse kidney prognosis and the presence of any sign of inflammation (MVI, tubullitis, TMA, and ATN). An important point to cite is that 37.5% of the patients with MVI ≥ 1 were retreated, and this treatment was associated with a lower rate of graft loss than those with persistent MVI who did not undergo an ABMR retreatment. However, these results have to be taken with caution since the sample size is small, and a potential selection bias cannot be ruled out.

Another significant element of this work is the presence of tubulitis in follow-up biopsies. The coexistence of a TCMR with an ABMR is a previously studied condition that significantly worsens the prognosis of ABMR in kidney transplantation (27, 28). Only one previous study has evaluated the tubulitis presence in follow-up biopsies after treatment of an ABMR, in which it was not associated with an increased risk of graft loss at 6 years (5). In contrast, we have observed that *t* ≥ 1, even without meeting criteria for TCMR diagnosis, was associated with substantially lower kidney graft survival.

Our findings suggest that persistent inflammation after aABMR treatment has a prognostic value, even when these inflammation signs did not fulfill any of the defined Banff categories and even when an initial improvement in kidney function is observed. This observation is consistent with the pathogenesis of chronic humoral damage, which is characterized by a sustained low-grade damage and glomerular basement membrane multilamination (transplant glomerulopathy, TG). This lesion has been associated with poor graft prognosis (29), as we have observed in the follow-up biopsies. This persistent glomerular inflammation may represent a sustained endothelial damage, over time, leading to TG.

This work has some limitations. Firstly, it is a retrospective study, and selection biases cannot be ruled out. The number of patients and events limit the statistical power, which prevents the performance of multivariate analyses. In Hospital Clinic de Barcelona, our guidelines include a follow-up biopsy after the ABMR treatment. However, the final decision for a kidney biopsy after ABMR treatment was based on clinical judgment. It should be noted that the rebiopsy periods were limited in time, and consistent to assess the response to treatment [mean time of 2 (4) months after treatment]. In addition, there were no differences in time to follow biopsy between the patients with and without persistent inflammation [2 (4.75) vs. 2 (4) months after treatment; *P* = 0.98, respectively].

Another important limitation that needs to be mentioned is the lack of a formal control group. Also, compliance is an important variable and could impact the analysis, although this is very difficult to address in a retrospective design. Moreover, the biological material to be examined with the molecular microscope (MMDx), which could provide additional information regarding the mechanisms of persistent inflammation (10), was not available.

Despite these limitations, we believe that the present study provides valuable information that reinforces the importance of follow-up biopsies after ABMR treatment, and revealed the persistence of MVI and tubulitis as markers of poor graft prognosis. As closing remarks, our study suggests the usefulness of systematically performing a follow-up biopsy after ABMR treatment, regardless of kidney graft function improvement after treatment, since the persistence of MVI or tubulitis seems to be associated with an increased risk of graft loss. Although it has not been established whether retreatment or other actions could modify this association, in this series, there was a better prognosis in the retreated patients with persistent inflammation than in the untreated patients (*p* = 0.048). More studies with a larger sample size are needed to confirm the findings of the present study, mainly focusing on evaluating the role of retreatment of patients with persistent inflammation in the control biopsy after treatment of ABMR.

## Data Availability Statement

The raw data supporting the conclusions of this article will be made available by the authors, without undue reservation.

## Ethics Statement

The studies involving human participants were reviewed and approved by Ethics Committee (CEIm) at Hospital Clinic de Barcelona. The patients/participants provided their written informed consent to participate in this study.

## Author Contributions

GP, EM-M, and JRo collected, analyzed, interpreted the data, and prepared the manuscript. JRí performed the statistical analysis and interpreted the data. PV-A, DC, IR, ML, JC, FC, NE, FO, JMC, BB-G, and FD interpreted the data and critically revised the manuscript. All authors contributed to the article and approved the submitted version.

## Funding

This study has been partially funded by Redes Temáticas De Investigación Cooperativa En Salud, REDINREN (RD16/0009/0023) by ISCIII-Subdirección General de Evaluación and Fondo Europeo de Desarrollo Regional (FEDER) Una manera de hacer Europa and Secretaria d'Universitats i Recerca and CERCA Programme del Departament d'Economia i Coneixement de la Generalitat de Catalunya (2017-SGR-1331).

## Conflict of Interest

The authors declare that the research was conducted in the absence of any commercial or financial relationships that could be construed as a potential conflict of interest.

## Publisher's Note

All claims expressed in this article are solely those of the authors and do not necessarily represent those of their affiliated organizations, or those of the publisher, the editors and the reviewers. Any product that may be evaluated in this article, or claim that may be made by its manufacturer, is not guaranteed or endorsed by the publisher.

## Abbreviations

ABMR: antibody-mediated rejection
aABMR: acute antibody-mediated rejection
AEMPS: Agencia Española de Medicamentos y Productos Sanitarios
ATN: acute tubular necrosis
CCI: Charlson comorbidity index
CI: confidence interval
DSAs: donor-specific antibodies
eGFR: estimated glomerular filtration rate
EMA: European Medicines Agency
FDA: Food and Drug Administration
HLA: human leukocyte antigen
HR: hazard ratio
IFTA: interstitial fibrosis and tubular atrophy
IQR: interquartile range
IVIg: intravenous immunoglobulin
MVI: microvascular inflammation
OR: odds ratio
PE: plasma exchange
SCr: Serum Creatinine
TCMR: T-cell mediated rejection
TG: transplant glomerulopathy
TMA: Thrombotic microangiopathy
UPCR: urine protein to creatinine ratio.
