# Supplementary material for: Influence of Persistent Inflammation in Follow-Up Biopsies After Antibody-Mediated Rejection in Kidney Transplantation
Source: Front Med (Lausanne). 2021 Nov 12;8:761919. doi: 10.3389/fmed.2021.761919 (PMC8632955; doi:10.3389/fmed.2021.761919)
Supplement: Supplementary file 1 [file Data_Sheet_1.pdf]

Influence of Persistent Inflammation in Follow-Up Biopsies After Antibody-Mediated Rejection in Kidney Transplantation

Gaston J Piñeiro[1,2], Enrique Montagud-Marrahi[1,2], José Ríos[3], Pedro Ventura-Aguiar[1,2], David Cucchiari[1,2], Ignacio Revuelta[1,2], Miquel Lozano[4], Joan Cid[4], Frederic Cofan[1], Nuria Esforzado[1], Eduard Palou[5], Federico Oppenheimer[1,2], Josep M Campistol[1,2,6], Beatriu Bayés-Genís[1,2], Jordi Rovira[2,6], Fritz Diekmann[1,2,6]

[1]Department of Nephrology and Kidney Transplantation, Hospital Clinic Barcelona, Spain. [2]Laboratori Experimental de Nefrologia i Trasplantament (LENIT), Institut d'Investigacions Biomèdiques August Pi i Sunyer (IDIBAPS), Barcelona, Spain.[3]Medical statistics platform, Institut d'Investigacions Biomèdiques August Pi i Sunyer (IDIBAPS), Barcelona, Spain. [4]Apheresis Unit, Department of Hemotherapy and Hemostasis, Hospital Clínic de Barcelona, Universitat de Barcelona, Barcelona, Spain. [5]Department of Immunology, Hospital Clínic de Barcelona, Universitat de Barcelona, Barcelona, Spain. [6]Red de Investigación Renal (REDINREN), Madrid, Spain.

SUPPLEMENARTY INFORMATION

Table S1. Demographic characteristics of patients with and without follow-up biopsy.

Table S2. Histological characteristics of the biopsy at ABMR diagnosis in patients with and without follow-up biopsy.

Table S3. Demographic characteristics of patients with and without persistent microvascular inflammation (MVI) in the follow-up biopsy.

Table S4. Histological characteristics of the biopsy at ABMR diagnosis in patients with and without persistent MVI.

Table S5. Death censored graft loss and MVI ≥ 1 in Follow-up Biopsy adjusted by histological characteristics.

Table S6. Death censored graft loss and histological variables adjusted by MVI ≥ 1 in Follow-up Biopsy.

Table S7. Histological characteristics and Death censored Graft loss, adjusted by time to rejection.

Table S1. Demographic characteristics of patients with and without follow-up biopsy.

	**Patients with Follow-up Biopsy (n = 90)**	**Patients without Follow-up Biopsy (n = 26)**	**P value**
Donor			
Age (years)	55.68 ± 13.61	51.19 ± 16.27	0.16
Gender (male)	41 (46)	18 (69)	0.03
DDKT	59 (66)	24 (92)	0.01
DCD	9 (15)	3 (13)	0.75
Recipient age (years)	49.58 ± 14.16	55 ± 12.71	0.08
Recipient gender (male)	55 (61)	14 (54)	0.51
Dialysis vintage (years)	2.5 [4.5]	4 [5]	0.66
Dialysis modality			0.98
Preemptive	15 (16)	4 (16)	
Hemodialysis	68 (76)	20 (76)	
Peritoneal dialysis	7 (8)	2 (8)	
ABMR < 1 year after KT (yes)	75 (83)	17 (65)	0.06
Hypertension (yes)	71 (79)	17 (65)	0.16
HCV (yes)	17 (19)	12 (46)	0.01
DM (yes)	15 (17)	5 (19)	0.77
Vasculopathy (yes)	16 (18)	3 (12)	0.45
ESKD etiology			0.41
ADPKD	12 (13)	5 (18)	
Urological	26 (29)	3 (12)	
Glomerulonephritis	19 (22)	7 (27)	
Diabetic nephropathy	9 (10)	3 (12)	
Nephroangiosclerosis	4 (4)	3 (12)	
Unknown	20 (22)	5 (19)	
Previous KT (any)	48 (53)	8 (31)	0.04
HLA mismatches	3.98 ± 1.62	4.50 ± 1.20	0.13
ABO incompatibility (yes)	12 (13)	4 (15)	0.79
Pre-transplant DSAs (any)	14 (16)	4 (15)	0.92

DSAs at ABMR diagnosis (any)	59 (70)	14 (54)	0.12
PRA > 50% (yes)	13 (14)	5 (19)	0.55
CF-CM at KT (positive)	15 (17)	2 (8)	0.13
Luminex at KT (positive)			
Class I	34 (39)	5 (19)	0.001
Class II	38 (42)	6 (23)	0.002
Induction Immunosuppression (yes)	88 (98)	24 (92)	0.18
Basiliximab	30 (33)	9 (35)	0.90
Rituximab	11 (12)	0 (0)	0.06
Thymoglobulin	54 (60)	14 (54)	0.65
Immunosuppression maintenance			
Tacrolimus	62 (69)	14 (54)	0.16
mTORi	33 (37)	8 (31)	0.58
Mycophenolate	72 (80)	20 (77)	0.73
Steroids	90 (100)	26 (100)	-
Need for dialysis at ABMR (yes)	26 (29)	6 (23)	0.56
ABMR treatment at diagnosis (yes)			
Plasma exchange	86 (96)	25 (96)	0.89
IVIg	18 (20)	4 (15)	0.60
Rituximab	80 (89)	21 (81)	0.28

Data are mean ± SD, median [IQR] or n (%), unless otherwise specified. ABMR, Antibody-mediated Rejection; KT, kidney transplantation; HCV, hepatitis C virus; DM, Diabetes Mellitus; ESKD, End-Stage Kidney Disease; ADPKD, Autosomal Dominant Polycystic Kidney Disease; HLA, Human Leucocyte Antigen; DSAs, Donor Specific Antibodies; PRA, Panel Reactive Antibodies; CF-CM, Flow Cytometry Crossmatch; mTORi, mTOR inhibitors; DDKT, Deceased Donor Kidney Transplantation; DCD, Donor after Cardiac Death; IVIg, Intravenous Immunoglobulin.

Table S2. Histological characteristics of the biopsy at ABMR diagnosis in patients with and without follow-up biopsy.

	**Patients with Follow-up Biopsy (n = 90)**	**Patients without Follow-up Biopsy (n = 26)**	**P value**
i	0.72 ± 0.92	0.54 ± 0.86	0.29
t	0.43 ± 0.78	0.5 ± 0.95	0.93
g	0.87 ± 0.97	0.77 ± 0.86	0.79
ptc	1.47 ± 0.96	1.3 ± 0.88	0.47
MVI (g + ptc)	2.33 ± 1.44	2.08 ±1.02	0.48
C4d	72 (80%)	23 (88.5%)	0.32
ATN	29 (32.2%)	6 (23.1%)	0.37
Vascular inflammation	0.21 ± 0.57	0.15 ± 0.54	0.41
Total inflammation	0.61 ± 0.84	0.27 ± 0.53	0.06
TMA	5 (5.6%)	1 (3.8%)	0.73
ci	0.66 ± 0.79	0.65 ±0.63	0.16
ct	0.64 ± 0.75	0.42 ± 0.64	0.68

Results are shown as mean ± SD for quantitative and qualitative variables. Qualitative variables, as C4d, are shown in absolute number and the percentage in brackets (% positive). i, interstitial inflammation; t, tubulitis; g, glomerulitis; ptc, peritubular capillaritis; ATN, acute tubular necrosis; atrophy; TMA, thrombotic microangiopathy; ci, interstitial fibrosis; ct, tubular atrophy.

Table S3. Demographic characteristics of patients with and without persistent microvascular inflammation (MVI) in the follow-up biopsy.

	**MVI persistence (n = 64)**	**Without MVI (n = 26)**	**P value**
Donor			
Age (years)	55.8 ± 13.7	55.4 ± 13.6	0.90
Gender (male)	27 (42)	14 (54)	0.36
DDKT	39 (61)	20 (77)	0,22
DCD	5 (13)	4 (20)	0.47
Recipient age (years)	48.8 ± 14.0	51.4 ± 14.7	0.44
Recipient gender (male)	38 (61)	16 (62)	0.96
Dialysis vintage (years)	4.5 [4.5]	3 [7]	0.43
Dialysis modality			0.43
Preemptive	12 (19)	3 (12)	
Hemodialysis	46 (72)	22 (85)	
Peritoneal dialysis	6 (9)	1 (4)	
ABMR < 1 year after KT (yes)	51 (80)	24 (92)	0.22
Hypertension (yes)	50 (78)	21 (81)	0.78
HCV (yes)	17 (11)	6 (23)	0.52
DM (yes)	8 (13)	7 (27)	0.10
Vasculopathy (yes)	10 (16)	6 (23)	0.40
ESKD etiology			0.65
ADPKD	10 (16)	2 (8)	
Urological	20 (31)	6 (23)	
Glomerulonephritis	13 (20)	6 (23)	
Diabetic nephropathy	5 (8)	4 (15)	
Nephroangiosclerosis	2 (3)	2 (8)	
Unknown	14 (22)	6 (23)	
Previous KT (any)	32 (50)	16 (62)	0.32
HLA mismatches	3.81 ± 1.74	4.39 ± 1.24	0.13
ABO incompatibility (yes)	10 (16)	2 (8)	0.32
Pre-transplant DSAs (any)	18 (28)	6 (23)	0.88
DSAs at ABMR diagnosis (any)	44 (76)	15 (58)	0.09
PRA > 50% (yes)	10 (16)	3 (12)	0.62
CF-CM at KT (positive)	12 (19)	3 (12)	0.34
Luminex at KT (positive)			
Class I	26 (42)	8 (31)	0.37
Class II	27 (42)	11 (42)	0.76
Induction Immunosuppression (yes)	63 (98)	25 (96)	0.51
Basiliximab	21 (33)	9 (35)	0.87
Rituximab	10 (16)	1 (4)	0.12
Thymoglobulin	39 (61)	15 (58)	0.78
Immunosuppression maintenance			
Tacrolimus	45 (70)	17 (65)	0.65
mTORi	24 (38)	9 (35)	0.80
Mycophenolate	51 (80)	21 (81)	0.91
Steroids	64 (100)	26 (100)	-
Need for dialysis at ABMR (yes)	18 (28)	8 (31)	0.80

ABMR treatment at diagnosis (yes)			
Plasma exchange	60 (94)	26 (100)	0.19
IVIg	13 (20)	5 (19)	0.91
Rituximab	58 (91)	22 (85)	0.41

Data are mean ± SD, median [IQR] or n (%), unless otherwise specified. ABMR, Antibody-mediated Rejection; KT, kidney transplantation; HCV, hepatitis C virus; DM, Diabetes Mellitus; ESKD, End-Stage Kidney Disease; ADPKD, Autosomal Dominant Polycystic Kidney Disease; HLA, Human Leucocyte Antigen; DSAs, Donor Specific Antibodies; PRA, Panel Reactive Antibodies; CF-CM, Flow Cytometry Crossmatch; mTORi, mTOR inhibitors; DDKT, Deceased Donor Kidney Transplantation; DCD, Donor after Cardiac Death; IVIg, Intravenous Immunoglobulin.

Table S4. Histological characteristics of the biopsy at ABMR diagnosis in patients with and without persistent MVI.

	**MVI persistence (n = 64)**	**Without MVI (n = 26)**	**P value**
i	0.77 ± 0.99	0.7 ± 0.9	0.822
t	0.42 ± 0.86	0.44 ± 0.75	0.71
g	0.65 ± 0.85	0.95 ± 1.01	0.22
ptc	1.34 ± 0.93	1.52 ± 0.98	0.44
MVI (g + ptc)	2 ± 1.33	2.47 ± 1.47	0.2
C4d	22 (84.6%)	50 (78.1%)	0.49
ATN	12 (46.2%)	17 (26.6%)	0.073
Vascular inflammation	0.38 ± 0.75	0.14 ± 0.47	0.037
Total inflammation	0.65 ± 0.8	0.59 ± 0.87	0.52
TMA	0	5 (7.8%)	0.14
ct	0.58 ± 0.76	0.67 ± 0.76	0.52
ci	0.64 ± 0.85	0.66 ± 0.78	0.89

Results are shown as mean ± SD for quantitative and qualitative variables. Qualitative variables, as C4d, are shown in absolute number and the percentage in brackets (% positive). i, interstitial inflammation; t, tubulitis; g, glomerulitis; ptc, peritubular capillaritis; ATN, acute tubular necrosis; atrophy; TMA, thrombotic microangiopathy; ci, interstitial fibrosis; ct, tubular atrophy.

Table S5. Death censored graft loss and MVI ≥ 1 in Follow-up Biopsy adjusted by histological characteristics.

Adjusted by	HR (95% CI)	P value
**Crude**	4.50 (1.35 – 15.0)	0.014
Acute cellular rejection (Yes)	3.51 (1.04 – 11.9)	0.044
Tubulitis ≥1 (Yes)	3.86 (1.15 – 12.9)	0.028
Tubulitis range	3.60 (1.08 – 12.0)	0.038
C4d + (Yes)	3.53 (1.04 – 12.0)	0.043
ATN (Yes)	5.71 (1.54 – 21.3)	0.009
Vascular inflammation ≥1 (Yes)	3.69 (1.09 – 12.5)	0.036
Total inflammation ≥1 (Yes)	3.60 (1.06 – 12.3)	0.04
Total inflammation range	3.76 (1.10 – 12.8)	0.034
TMA (Yes)	2.90 (0.84 – 10.0)	0.092
IFTA ≥1 (Yes)	3.56 (1.06 – 12.0)	0.04
IFTA range	3.43 (1.01 – 11.6)	0.047
Transplant Glomerulopathy ≥1 (Yes)	3.24 (0.94 – 11.2)	0.062

ATN, acute tubular necrosis; atrophy; TMA, thrombotic microangiopathy; IFTA: interstitial fibrosis and tubular atrophy.

Table S6. Death censored graft loss and histological variables adjusted by MVI ≥ 1 in Follow-up Biopsy.

	HR (95% CI)	P value
Acute cellular rejection (Yes)	1.32 (0.50 – 3.49)	0.581
Tubulitis ≥1 (Yes)	1.36 (0.59 – 3.12)	0.472
Tubulitis range	1.85 (1.04 – 3.29)	0.035
C4d + (Yes)	1.16 (0.39 – 3.50)	0.790
ATN (Yes)	9.19 (2.52 – 33.5)	0.001
Vascular inflammation ≥1 (Yes)	1.32 (0.38 – 4.53)	0.661
Total inflammation ≥1 (Yes)	1.25 (0.79 – 1.98)	0.338
Total inflammation range	1.76 (0.80 – 3.86)	0.158
TMA (Yes)	0.65 (0.09 – 4.85)	0.675
IFTA ≥1 (Yes)	1.56 (0.74 – 3.30)	0.247
IFTA range	1.46 (1.02 – 2.09)	0.040
Transplant Glomerulopathy ≥1 (Yes)	2.66 (1.13 – 6.24)	0.025

ATN, acute tubular necrosis; atrophy; TMA, thrombotic microangiopathy; IFTA: interstitial fibrosis and tubular atrophy.

Table S7. Histological characteristics and Death censored Graft loss, adjusted by time to rejection.

(A) Biopsy at ABMR diagnosis	Crude OR (95% CI)	Crude P value	Adjusted by Time to rejection OR (95% CI)	Adjusted by Time to rejection P value
Acute cellular rejection (Yes)	2.21 (1.07 – 4.58)	0.033	2.15 (1.03 – 4.50)	0.042
Tubulitis (Yes)	1.85 (1.30 – 2.65)	0.001	1.52 (1.03 – 2.23)	0.033
Tubulitis range	2.10 (1.04 – 4.26)	0.039	1.44 (0.68 – 3.05)	0.338
Glomerulitis	0.92 (0.64 – 1.34)	0.678	0.94 (0.64 – 1.38)	0.747
Peritubular Capilaritis (Yes)	1.56 (1.09 – 2.24)	0.016	2.99 (1.26 – 7.09)	0.013
MVI (g + ptc) ≥1 (Yes)	1.19 (0.93 – 1.51)	0.161	1.15 (0.89 – 1.49)	0.279
C4d + (Yes)	1.23 (0.42 – 3.57)	0.709	1.36 (0.46 – 3.97)	0.576
ATN (Yes)	0.80 (0.38 – 1.67)	0.550	1.06 (0.49 – 2.28)	0.879
vascular inflammation ≥1 (Yes)	0.76 (0.36 – 1.63)	0.486	0.79 (0.36 – 1.70)	0.544
Total inflammation ≥1 (Yes)	1.07 (0.66 – 1.74)	0.782	0.90 (0.56 – 1.44)	0.659
TMA (Yes)	0.82 (0.11 – 6.02)	0.846	0.93 (0.13 – 6.86)	0.946
IFTA ≥1 (Yes)	1.62 (1.09 – 2.40)	0.017	1.35 (0.89 – 2.06)	0.161
IFTA range	2.30 (1.16 – 4.56)	0.017	1.87 (0.92 – 3.77)	0.083

(B) Follow-up Biopsy	Crude OR (95% CI)	Crude P value	Adjusted by Time to rejection OR (95% CI)	Adjusted by Time to rejection P value
Acute cellular rejection (Yes)	1.54 (0.58 – 4.03)	0.384	1.46 (0.56 – 3.84)	0.441
Tubulitis ≥1 (Yes)	1.92 (1.09 – 3.36)	0.023	1.68 (0.95 – 2.97)	0.074
Tubulitis range	2.88 (1.24 – 6.69)	0.014	2.57 (1.09 – 6.02)	0.030
Glomerulitis ≥1 (Yes)	1.06 (0.72 – 1.54)	0.774	1.10 (0.74 – 1.66)	0.630
Peritubular Capilaritis ≥1 (Yes)	2.99 (1.27 – 7.07)	0.012	2.99 (1.26 – 7.09)	0.013
MVI (g + ptc) ≥1 (Yes)	4.50 (1.35 – 15.0)	0.014	3.61 (1.07 – 12.2)	0.039
C4d + (Yes)	1.77 (0.60 – 5.19)	0.297	1.87 (0.63 – 5.53)	0.257
ATN (Yes)	4.56 (1.53 – 13.6)	0.006	4.30 (1.39 – 13.3)	0.011
vascular inflammation ≥1 (Yes)	0.11 (0.00 – 663.7)	0.665	0	0.999
Total inflammation ≥1 (Yes)	1.58 (1.02 – 2.46)	0.040	1.27 (0.80 – 2.01)	0.312
TMA (Yes)	3.70 (1.50 – 9.15)	0.005	4.60 (1.81 – 11.7)	0.001
IFTA ≥1 (Yes)	1.62 (1.14 – 2.32)	0.008	1.45 (1.01 – 2.08)	0.041
IFTA range	2.30 (0.87 – 6.09)	0.093	2.36 (0.89 – 6.28)	0.085
Transplant Glomerulopathy ≥1 (Yes)	2.24 (1.33 – 3.77)	0.003	1.92 (1.10 – 3.34)	0.022
Transplant Glomerulopathy range	4.39 (1.96 – 9.83)	0.000	3.26 (1.38 – 7.66)	0.007

MVI, microvascular inflammation; ATN, acute tubular necrosis; atrophy; TMA, thrombotic microangiopathy; IFTA: interstitial fibrosis and tubular atrophy.
